# Systematic Investigation of mRNA *N*^6^-Methyladenosine Machinery in Primary Prostate Cancer

**DOI:** 10.1155/2020/8833438

**Published:** 2020-11-12

**Authors:** Mei Jiang, Yali Lu, Dongxia Duan, Hongxiang Wang, Gaoya Man, Ching Kang, Kesaier Abulimiti, Yao Li

**Affiliations:** ^1^Shanghai Tenth People's Hospital, Tongji University School of Medicine, Shanghai 200072, China; ^2^State Key Laboratory of Genetic Engineering, Shanghai Engineering Research Center of Industrial Microorganisms, School of Life Science, Fudan University, Shanghai 200433, China; ^3^Shanghai Key Laboratory of Psychotic Disorders, Shanghai Mental Health Center, Shanghai Jiao Tong University School of Medicine, Shanghai 201108, China; ^4^Department of Neurosurgery, Shanghai Changzheng Hospital, Second Military Medical University, Shanghai 200003, China; ^5^Hepatobiliary and Vascular Surgery, Tengzhou Central People's Hospital, Tengzhou 277500, China

## Abstract

**Background:**

Appreciable findings have pointed out pivotal roles of *N*^6^-methyladenosine (m^6^A) machinery in cancer onset and progression. However, limited efforts have been directed towards relevant research in the prostate cancer area.

**Methods:**

A PubMed search was conducted to acquire components of the mRNA m^6^A machinery. Multiomics integration was performed to systematically investigate the mRNA m^6^A machinery in primary prostate cancer. Furthermore, RNA interference assays of two prognostic m^6^A readers EIF3D and HNRNPA2B1 were conducted to explore m^6^A dependence of their functions in prostate cancer cell proliferation and migration.

**Results:**

A total of 41 mRNA m^6^A regulators have been identified to date. A small degree of copy number aberrations and an extremely low frequency of somatic mutations were observed in the regulators across prostate tumors. Enrichment of CpG sites and extensive changes of DNA methylation in the m^6^A machinery were also found. Impact of copy number variation on m^6^A regulator expression was stronger than that of DNA methylation disturbance. Furthermore, our study identified a set of m^6^A regulators related to clinical features and/or survival which were largely m^6^A-binding proteins. The translation initiation factor subunit EIF3D and the splicing factor HNRNPA2B1 can be independent prognostic factors which may contribute to retardation and promotion of cancer progression, respectively, through affecting cancer-related processes such as cell cycle. Moreover, *in vitro* assays demonstrated that m^6^A impacted the EIF3D and HNRNPA2B1 roles in proliferation and migration of prostate cancer cells.

**Conclusions:**

Our report systematically described molecular features of the mRNA m^6^A machinery and their potential roles in primary prostate cancer. Knowledge gained from this work may pave the way for further studies on the m^6^A system in prostate cancer.

## 1. Introduction

Emerging evidence highlights roles of the epitranscriptome in development and disease [1–4]. So far, over 150 distinct types of RNA modifications have been discovered [5], and *N*^6^-methyladenosine (m^6^A) is the most prevalent modification in eukaryotic mRNAs and noncoding RNAs [1, 2].

The cellular m^6^A machinery consists of writers, erasers, and readers [1–3]. The first two members are responsible for deposition and removal of a methyl group at the nitrogen-6 position of the RNA adenosine base, respectively. The METTL3-METTL14 methyltransferase complex is required for the formation of the majority of m^6^A modifications in mRNAs [6], comprising the METTL3 catalytic subunit and other cofactors [1–3]. The other writer METTL16 catalyzes m^6^A modifications in noncoding RNAs and a small number of mRNAs [7]. To date, only two erasers have been identified including FTO and ALKBH5 [1–4]. Demethylase activity of FTO is not restricted to m^6^A [8, 9] and exhibits very limited effects on m^6^A stoichiometry [9, 10]. ALKBH5 is normally enriched in the testis, and its mediated m^6^A demethylation is crucial to spermatogenesis [11].

Effects of m^6^A methylation on RNA fate are exerted by its readers which recognize and bind to m^6^A sites [1–3]. Currently, the best-characterized m^6^A readers include YTH domain-containing proteins and the eukaryotic translation initiation factor eIF3 [1–3]. YTHDF2 contributes to m^6^A-directed mRNA destabilization through conducting its bound RNAs to processing bodies [12]. YTHDC1 can recruit the splicing factor SRSF3 to m^6^A sites thereby promoting exon inclusion [13]. The eIF3 complex is required for m^6^A-dependent translation enhancement [14], which may involve YTHDF1 in some cases [15].

Implications of METTL3, METTL14, FTO, ALKBH5, and YTHDF2 have been demonstrated in several types of cancer such as acute myeloid leukemia, hepatocellular cancer, and glioblastoma [3]. However, systematic studies on the m^6^A machinery are still limited in the cancer field [16–18]. Furthermore, we lack knowledge regarding the m^6^A system in prostate carcinogenesis according to our PubMed search, even including the METTL3-METTL14 core subunits. In the present study, we integrated genomic, epigenomic, transcriptomic, proteomic, and clinicopathologic profiles of primary prostate tumors from The Cancer Genome Atlas (TCGA, https://portal.gdc.cancer.gov) to systematically decipher mRNA m^6^A writers, erasers, and readers in prostate cancer. Molecular alterations of the mRNA m^6^A regulators were investigated in a variety of levels. Furthermore, we identified prognostic mRNA m^6^A regulators for prostate cancer and explored their potential roles in prostate carcinogenesis.

## 2. Materials and Methods

### 2.1. Data Accession and Processing

To acquire components of the mRNA m^6^A machinery, a PubMed search was conducted using the following keywords: “m6A”[TIAB] OR “m(6)A”[TIAB] OR “N6-methyladenosine”[TIAB] OR “N(6)-methyladenosine”[TIAB]. Retrieved documents were subjected to a manual review.

Multiomics datasets of 498 primary prostate tumors and 52 matched normal tissues were obtained through TCGAbiolinks [19], including copy number variations, genomic mutations, CpG methylation, gene , isoform, and protein expression, clinicopathology, and molecular subtypes. The data were generated by The Cancer Genome Atlas (TCGA, https://portal.gdc.cancer.gov). When estimating gene expression, mapped fragments per kilobase per million (FPKM) values were calculated based on RNA-seq read counts using edgeR [20], then log2 transformed, and finally *Z*-score scaled. We called the standardized values zFPKM which estimated relative gene expression levels across samples.

### 2.2. Survival Analyses

Patients were grouped into two populations above and below the median. Log-rank tests provided by the R package survival [21] were used to compare the survival of patient groups, and Kaplan–Meier survival curves were plotted by using the R package survminer. Hazard ratios were estimated by either the univariate Cox proportional hazard regression model or the multivariate LASSO Cox regression model. The Wald test was used to calculate the significance of the univariate Cox regression model (*p* value < 0.05). The univariate and multivariate Cox regressions were accomplished by survival [21] and glmnet [22], respectively.

The risk score of the selected RNA m^6^A regulators was calculated in the following way:

(1) $$\begin{array}{c} \text{Risk}\text{score}=\overset{n}{\underset{i=1}{\sum }}L_{i}*X_{i}, \end{array}$$

where *L*_*i*_ is the coefficient of the *i*th m^6^A regulator calculated by the LASSO Cox regression and *X*_*i*_ is the zFPKM of the *i*th m^6^A regulator.

### 2.3. Statistical Analyses

Two-tailed *t*-tests were used to compare two groups of samples. To compare three or more groups, one-way ANOVA was performed, followed by Tukey's HSD tests for multiple pairwise comparisons if the ANOVA tests were significant (*p* value < 0.05).

### 2.4. *In Silico* Functional Analyses of EIF3D and HNRNPA2B1

The TCGA prostate cancer project has quantified a total of 205 proteins which do not include METTL3, EIF3D, and HNRNPA2B1. The Pearson correlation was calculated between the expression level of the TCGA-assayed proteins and the *EIF3D* transcript abundance. Proteins positively correlated with *EIF3D* (falsediscoveryrate(FDR) < 0.1) were regarded to be translationally controlled by EIF3D. Overrepresentation analysis was performed on the EIF3D-regulated proteins using clusterProfiler [23]. The Gene Expression Omnibus database (GEO, https://www.ncbi.nlm.nih.gov/geo/) was queried about EIF3D-bound transcripts using the keyword EIF3D, and three data series of crosslinking immunoprecipitation sequencing (CLIP-seq) were collected, including GSE73405, GSE65004, and GSE91432. To examine dependence of the EIF3D regulation on m^6^A modifications, the Pearson correlation was calculated between expression levels of the EIF3D-regulated proteins and the *METTL3* transcript abundance (FDR < 0.1). Moreover, to determine whether those EIF3D-regulated transcripts contain preferred sites for m^6^A modifications, an inquiry was launched into the RMBase database [24] which provides a map of experimentally validated RNA modifications.

The Pearson correlation was calculated between the abundance of all transcript isoforms and gene expression levels of either *METTL3* or *HNRNPA2B1*. For genes showing positive correlation (coefficient > 0.3 and FDR < 0.05) with both *METTL3* and *HNRNPA2B1* and containing candidate sites for m^6^A modifications based on the RMBase database, their alternative splicing might involve HNRNPA2B1 and m^6^A modifications. Overrepresentation analysis was performed on the m^6^A-dependent HNRNPA2B1-regulated genes using clusterProfiler. In addition, three CLIP-seq datasets of HNRNPA2B1 binding were obtained from the GEO database using the keyword HNRNPA2B1, including GSE103165, GSE70061, and GSE34996.

### 2.5. Data Visualization

Visualization tools included Cytoscape [25] and R packages such as ggplot2 [26], ggsignif, ComplexHeatmap [27], and clusterProfiler [23].

### 2.6. Cell Culture and Transfection

The human prostate cancer cell line C4-2B (American Type Culture Collection CRL-3315) was cultured in DMEM (HyClone) supplemented with 10% FBS (Biological Industries) at 37°C with 5% CO_2_.

Transfection with siRNAs was performed using HilyMax (Dojindo). Cells were harvested six hours following transfection and used for studying effects of gene knockdown. Negative control siRNAs and siRNAs targeting *ALKBH5*, *EIF3D*, and *HNRNPA2B1* were purchased from GenePharma (Shanghai, China). Sequences are listed in Supplementary Table S1.

### 2.7. Quantitative RT-PCR

Total RNA was extracted using TRIzol (Invitrogen) and reversely transcribed using NovoScript 1^st^ Strand cDNA Synthesis SuperMix (Novoprotein). Quantitative PCR was performed in triplicate using AceQ qPCR SYBR Green Master Mix (Vazyme Biotech). *Actin* was used as the reference gene. Primer sequences are listed in Supplementary Table S2.

### 2.8. Cell Proliferation Assay

Transfected cells were seeded at 4000 cells/well into 96-well plates. After 0 d, 1 d, 2 d, 3 d, and 4 d cell culture, 10 *μ*L of Cell Counting Kit-8 (Dojindo) was added to each well, and cells were incubated at 37°C for another 2 hours. Each time point was repeated six times. The viable cell number was quantified by measuring the absorbance at 450 nm normalized to the absorbance at 615 nm.

### 2.9. Cell Migration Assay

2 × 10^4^ transfected cells were suspended in DMEM with 1% FBS and added to each upper chamber of 24-well Transwell plates (Corning). DMEM containing 5% FBS was added to the bottom chamber. After 72 h incubation at 37°C in a 5% CO_2_ atmosphere, cells were fixed with methanol, then stained with DAPI, and counted in three microscopic fields. Each experiment was performed in triplicate.

## 3. Results

### 3.1. Genomic and Epigenomic Alterations of mRNA m^6^A Regulators in Prostate Cancer

**Figure 1:**
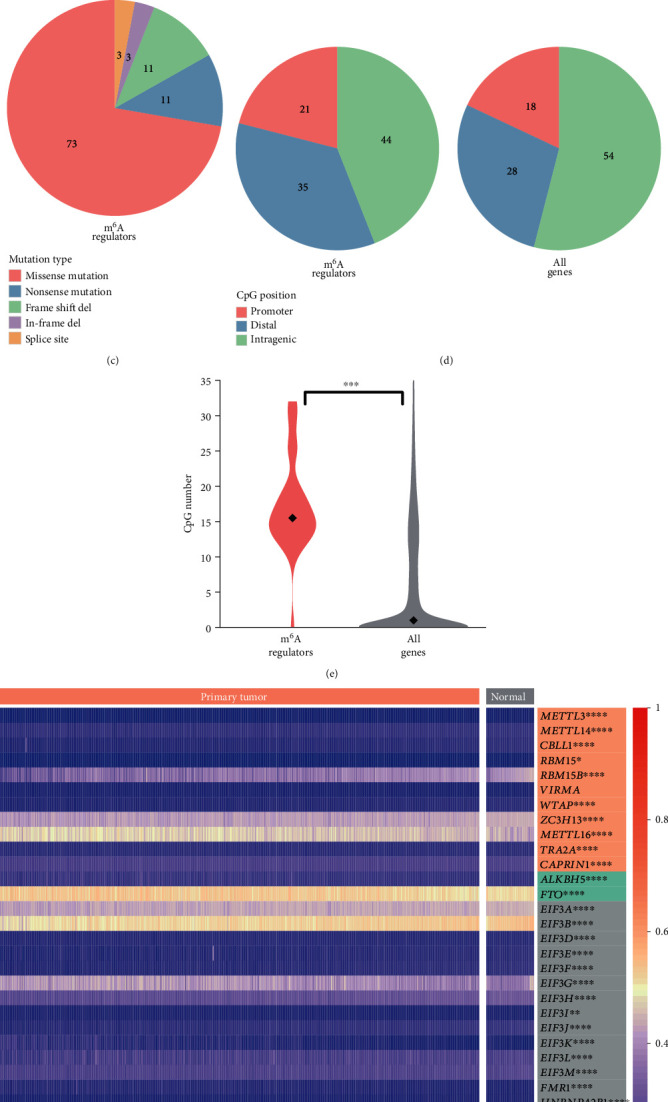
Genomic, epigenomic, and transcriptomic profiles of the mRNA m^6^A regulators in prostate cancer. (a) RNA m^6^A writers, erasers, and readers and their interaction. The association confidence was calculated by STRING [52] based on the STRING's collection of experiments, databases, and coexpression data. The higher the score is, the tighter the association is. The protein interactions highlighted in blue were manually annotated according to previous studies [53, 54]. The arrowheads indicate the regulators not expressed in either normal prostates or prostate tumors. (b) Copy number variation (CNV) in the coding regions. The CNV was categorized by log2 copy number change: amplification (1, +∞), gain (0.3, 1), shallow deletion (-1, -0.3), and deep deletion (−∞, -1). (c) The most frequent mutation was missense mutation. (d) Distribution of CpG methylation sites in regulatory regions and gene bodies of the m^6^A regulators was similar to that of other genes. Promoter: -100 bp to +100 bp from transcription start site; distal: more than 100 bp upstream of the transcription start site. (e) Significantly more CpG methylation sites were observed in the m^6^A regulators. (f) Genomic methylation and (g) transcriptomic profiles in normal and cancerous prostates. Each row in (f) and (g) represents mean CpG methylation beta-values and zFPKM values of a regulator across all samples, respectively. Significance of difference between prostate tumors and normal prostates is marked with one or more asterisks right beside genes. RNA m^6^A writers, erasers, and readers are highlighted in orange, green, and grey, respectively. ∗ denotes *p* value < 0.05, ∗∗ denotes *p* value < 0.01, ∗∗∗ denotes *p* value < 0.001, and ∗∗∗∗ denotes *p* value < 0.0001.

A PubMed query retrieved 1740 publications regarding *N*^6^-methyladenosine, and a total of 41 mRNA m^6^A regulators have been identified to date (Figure 1(a) and Supplementary Table S3) which fall into three classes: writers, erasers, and readers [1–3]. The writers mainly include the METTL3-METTL14 complex and METTL16 [1–3] (Figure 1(a) and Supplementary Table S3). The complex consists of METTL3, METTL14, WTAP, VIRMA, RBM15, RBM15B, ZC3H13, and CBLL1 [1–3], which is a major player in mRNA m^6^A methylation [6]. The erasers include FTO and ALKBH5 [1–3] (Figure 1(a) and Supplementary Table S3). Diverse readers recognize m^6^A sites (Figure 1(a) and Supplementary Table S3), such as YTH proteins (YTHDC1, YTHDC2, YTHDF1, YTHDF2, and YTHDF3), eIF3, and heterogeneous nuclear ribonucleoproteins (HNRNPA2B1 and HNRNPC) [1–3]. eIF3 comprises 13 subunits (EIF3A to EIF3M) [28]. All mRNA m^6^A regulators were detected on the mRNA level in prostate tumors and normal prostates except *IGF2BP1* and *IGF2BP3* which were excluded from subsequent analyses.

Detectable copy number changes were observed in the protein coding regions of all mRNA m^6^A regulators but usually at a low level (Figure 1(b)). The vast majority of the changes were gains and shallow deletions, and the frequencies were commonly below 10%. Mutations were rare in m^6^A regulators (Supplementary Table S4), more than 70% of which were missense (Figure 1(c)). In fact, prostate tumors carry lower somatic mutation burdens compared with other types of solid tumors [29].

DNA methylation occurs predominantly at CpG dinucleotides [4]. CpG methylation and demethylation influence the transcription level of neighboring genes, and its deregulation is a seminal hallmark of cancer cells [4]. According to the genomic DNA methylation profiling of prostate tumors and normal prostates, CpG sites largely resided in gene bodies (54%), followed by upstream distal regulatory regions (28%) and promoters (18%), which was the same case for mRNA m^6^A regulators (Figure 1(d)). Intriguingly, overall density of CpG sites in m^6^A regulators was far above the average level (Figure 1(e)). The CpG enrichment was found in both regulatory regions and gene bodies of m^6^A regulators (Supplementary Figures S1A–S1C). Additional investigation revealed altered DNA methylation levels in prostate tumors compared with normal prostates for all m^6^A regulators except *VIRMA* and *EIF3C*, the latter one of which was completely devoid of CpG methylation (Figure 1(f) and Supplementary Excel S1). Increases were observed in approximate one-fourth of the regulators including both erasers (Supplementary Figures S2A–S2C and Excel S1).

### 3.2. Transcriptomic Changes of mRNA m^6^A Regulators in Prostate Cancer

30 out of 39 mRNA m^6^A regulators exhibited altered expression in prostate cancer (Figure 1(g) and Supplementary Figure S3 and Excel S2). Four and two components of the METTL3-METTL14 methylation complex were upregulated and downregulated, respectively (Supplementary Figure S3 and Excel S2). Overexpression of the m^6^A methyltransferase METTL3 has been reported in a variety of prostate cancer cell lines, which promotes cell proliferation, survival, and invasion [30]. Both erasers decreased in prostate cancer (Supplementary Figure S3 and Excel S2). Overwhelming upregulation of writers, especially METTL3, and downregulation of erasers hinted an increase in mRNA m^6^A modifications in prostate cancer, which was indirectly confirmed by the observations that most of the readers were upregulated in prostate cancer (Supplementary Figure S3 and Excel S2). In agreement with our speculation, elevated RNA m^6^A levels have been reported in prostate cancer cell lines [30] and castration-resistant prostate cancer samples [31]. We also noticed that expression of the mRNA m^6^A regulators was generally more variable in tumors than in normal tissues (Supplementary Figure S3), again indicating dysregulation of m^6^A machinery in tumors.

**Figure 2:**
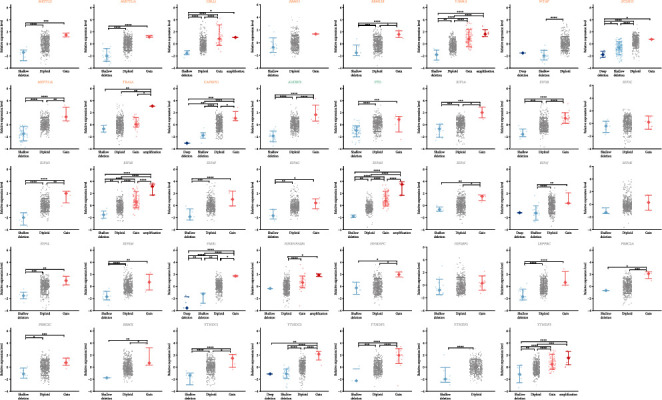
Impact of gene copy number on the expression level of the mRNA m^6^A regulators in prostate cancer. Writers, erasers, and readers are marked in orange, green, and grey, respectively. ∗ denotes *p* value < 0.05, ∗∗ denotes *p* value < 0.01, ∗∗∗ denotes *p* value < 0.001, and ∗∗∗∗ denotes *p* value < 0.0001.

**Figure 3:**
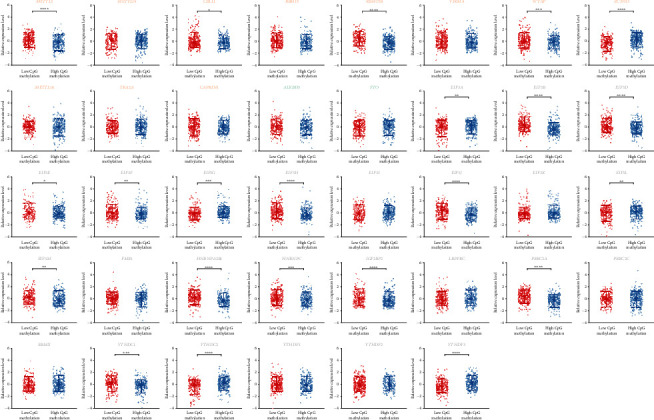
Effect of genomic methylation level on transcript abundance of the mRNA m^6^A regulators in prostate cancer. Writers, erasers, and readers are marked in orange, green, and grey, respectively. ∗ denotes *p* value < 0.05, ∗∗ denotes *p* value < 0.01, ∗∗∗ denotes *p* value < 0.001, and ∗∗∗∗ denotes *p* value < 0.0001.

We further investigated the impact of the genomic and epigenomic alterations on the gene transcription levels of mRNA m^6^A regulators. Due to rare mutation events, their effects were not explored in the current study. Copy number variations effectively changed the expression of most m^6^A regulators (90%, Figure 2). As expected, gains of gene copies increased transcription while losses reduced it. The impact of DNA methylation on m^6^A regulators seemed limited (Figure 3). CpG methylation led to an expression decline of 16 genes, consistent with its implication in transcription repression [4]. However, it did not change and even occasionally increased transcription of other regulators. 15 regulators were affected by both copy number variation and DNA methylation status, while *RBM15*, *EIF3C*, and *EIF3K* were affected by neither. On the whole, the influence of copy number variations was more extensive than that of DNA methylation on mRNA m^6^A regulators.

### 3.3. Association of mRNA m^6^A Regulators with the Clinical and Molecular Features of Prostate Cancer

**Figure 4:**
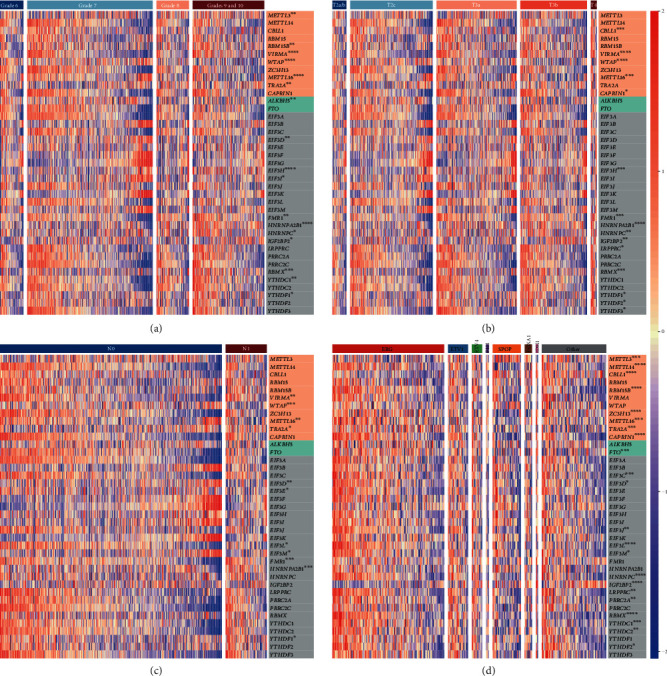
Relevance of the mRNA m^6^A regulator expression to malignancy degree and molecular subtypes in prostate cancer. (a–d) *Z*-score-transformed FPKM values in prostate tumors of Gleason grades 6 to 10, T stages T2a to T4, N stages N0 to N1, and different molecular subtypes. Samples are displayed in increasing order of aggressiveness from left to right in (a)–(c). RNA m^6^A writers, erasers, and readers are highlighted in orange, green, and grey backgrounds, respectively. ∗ denotes *p* value < 0.05, ∗∗ denotes *p* value < 0.01, ∗∗∗ denotes *p* value < 0.001, and ∗∗∗∗ denotes *p* value < 0.0001.

We then investigated the clinical significance of mRNA m^6^A regulators for prostate cancer. There are two common systems in clinical practice to determine the malignancy of prostate cancer, including Gleason score grading and TNM staging [32]. The Gleason score is used to grade abnormality of the cells from a biopsy, ranging from 6 to 10. The T, N, and M categories of the TNM staging describe size and extent of the primary tumor, spread of cancer to nearby lymph nodes, and distant metastasis, respectively. In both systems, a larger number indicates a more aggressive phenotype. Expression variations among Gleason score groups were detected in sixteen regulators, including elevation of four writers and eight readers and decline of two writers, one eraser, and one reader in high-grade tumors (Figure 4(a) and Supplementary Figure S4 and Excel S3). Thirteen and eleven regulators were differentially expressed among T and N stages, respectively (Figures 4(b) and 4(c) and Supplementary Figure S5 and Excel S4). In advanced T stages, two writers and nine readers were increased while two writers were decreased. In advanced N stages, two writers and three readers were increased while two writers and four eIF3 subunits were decreased. Due to only a few tumors of the advanced M stage (three M1 samples), analysis of M stages was not taken. *VIRMA*, *TRA2A*, *EIF3H*, *FMR1*, *HNRNPA2B1*, *HNRNPC*, *IGF2BP2*, *RBMX*, and *YTHDF1* were upregulated in both high grade and advanced stage, while *WTAP*, *METTL16*, and *EIF3D* were downregulated in both.

Based on genomic profiles, prostate cancer can be divided into seven molecular subtypes which bear distinct oncogenic drivers including fusions with ETS family members (*ERG*, *ETV1*, *ETV4*, and *FLI1*) and mutations in *SPOP*, *FOXA1*, and *IDH1* [29]. Expression of more than half of the regulators differed among molecular subtypes (Figure 4(d) and Supplementary Figure S6 and Excel S5); thus, those molecular subtypes manifested distinct expression profiles of m^6^A regulators.

### 3.4. Prognostic Values of mRNA m^6^A Regulators for Prostate Cancer

**Figure 5:**
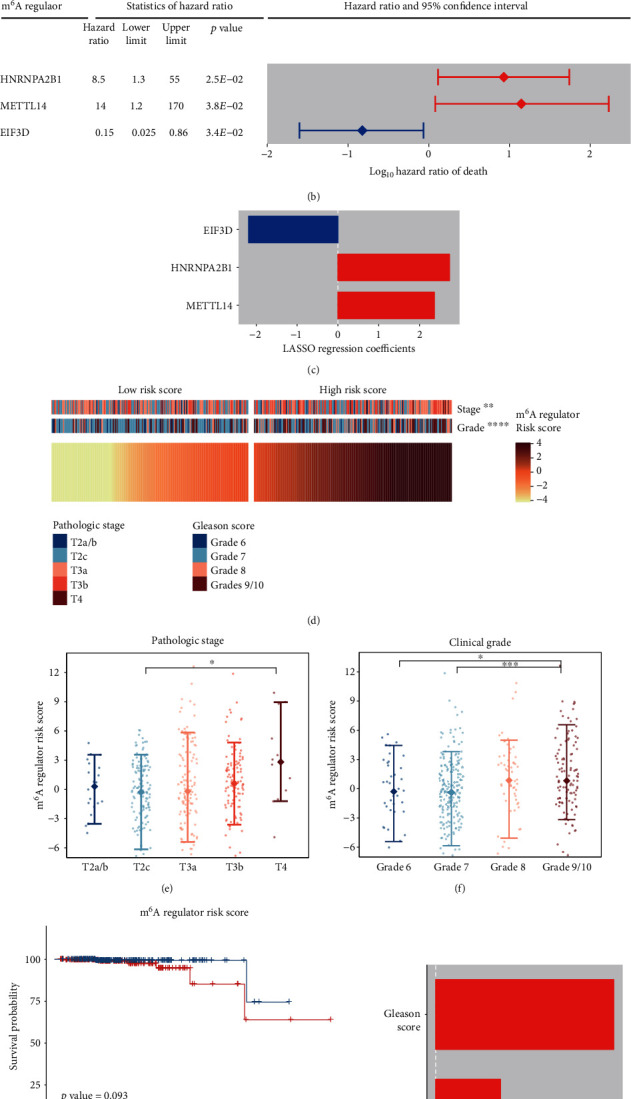
Prognostic values of the mRNA m^6^A regulators for death. (a) Overall survival-relevant regulators revealed by Kaplan–Meier survival analyses (*p* value < 0.1). (b) Hazard ratios and 95% confidence intervals of hazard ratios of the regulators for death (*p* value < 0.05). (c) LASSO Cox regression coefficients of the regulators for death. In (b) and (c), the m^6^A regulators prone to good and poor prognosis are colored in blue and red, respectively. (d) Clinicopathological differences between groups of low and high risk scores. (e, f) Risk scores were higher in tumors of advanced stage/grade. (g) The risk score was moderately associated with the overall survival (*p* value < 0.1). (h) LASSO Cox regression coefficients of the risk factors for the overall survival. ∗ denotes *p* value < 0.05, ∗∗ denotes *p* value < 0.01, ∗∗∗ denotes *p* value < 0.001, and ∗∗∗∗ denotes *p* value < 0.0001.

Innovative diagnostics has facilitated therapeutic decision-making with prostate cancer, such as development of molecular tools [32]. To explore prognostic values of mRNA m^6^A regulators, Kaplan–Meier survival analyses were first performed. Significant association with overall survival was found in *EIF3D* and *HNRNPA2B1* (*p* value <0.05), and moderate association was found in *RBM15* (0.05 < *p*value < 0.1) (Figure 5(a)). These genes were indicative of a poor prognosis except *EIF3D*. Moreover, univariate Cox regression analyses for death revealed that *HNRNPA2B1* and *METTL14* were disadvantageous factors while *EIF3D* was a beneficial one (*p* value < 0.05, Figure 5(b)). To better predict the clinical outcome, the LASSO Cox regression model was applied to remove regulators with minor effects on the survival and build a prognostic signature. *HNRNPA2B1*, *METTL14*, and *EIF3D* could independently contribute to the overall survival (Figure 5(c)) and were used to construct the prognostic signature for death. Subsequently, risk scores of patients were calculated, which took expression levels of the prognostic signature constituent m^6^A regulators and the LASSO regression coefficients into account. It is not surprising that higher risk scores were frequently observed in the patients carrying prostate tumors of advanced stage and high grade (Figures 5(d)–5(f)). Furthermore, the risk score of the m^6^A regulator signature could be a prognostic factor for death according to the Kaplan–Meier survival analysis (Figure 5(g)), which was confirmed by the multivariate Cox regression analysis (Figure 5(h)).

**Figure 6:**
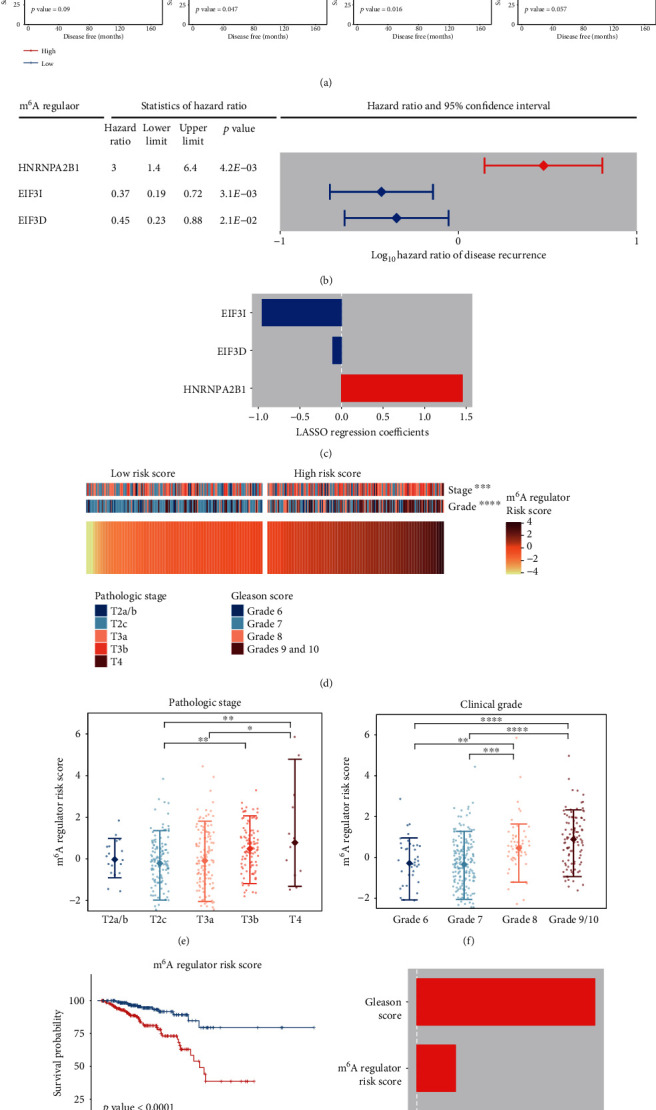
Prognostic values of the mRNA m^6^A regulators for cancer relapse. (a) Recurrence-free survival-relevant regulators according to Kaplan–Meier survival analyses (*p* value < 0.1). (b) Hazard ratios and 95% confidence intervals of hazard ratios of the regulators for the disease recurrence (*p* value < 0.05). (c) LASSO Cox regression coefficients of the regulators for the recurrence-free survival. In (b) and (c), the m^6^A regulators associated with the favorable and poor survival outcome are colored in blue and red, respectively. (d) Clinicopathological differences between groups of low and high risk scores. (e, f) Risk scores were higher in tumors of advanced stage/grade. (g) Worse disease-free survival was observed in the patients of higher risk scores (*p* value < 0.0001). (h) LASSO Cox regression coefficients of the risk factors for the recurrence-free survival. ∗ denotes *p* value < 0.05, ∗∗ denotes *p* value < 0.01, ∗∗∗ denotes *p* value < 0.001, and ∗∗∗∗ denotes *p* value < 0.0001.

We also investigated prognostic values of mRNA m^6^A regulators for cancer relapse. Based on Kaplan–Meier survival analyses, *TRA2A*, *FTO*, *EIF3I*, and *HNRNPA2B1* were significantly linked to the disease-free survival (*p* value < 0.05), and moderate association was observed in *METTL16*, *EIF3D*, and *YTHDC2* (0.05 < *p*value < 0.1) (Figure 6(a)). Among these cancer relapse-relevant genes, *METTL16*, *FTO*, *EIF3D*, and *EIF3I* were good prognostic factors, while *TRA2A*, *HNRNPA2B1*, and *YTHDC2* were bad ones. Univariate and multivariate Cox regression analyses affirmed the prognostic values of *EIF3I*, *EIF3D*, and *HNRNPA2B1*, the first two of which implied a low risk of recurrence (Figures 6(b) and 6(c)). Higher risk scores of *EIF3I*, *EIF3D*, and *HNRNPA2B1* were found in the patients with advanced tumors (Figures 6(d)–6(f)). Furthermore, Kaplan–Meier survival and LASSO Cox regression analyses suggested that the risk score of *EIF3I*, *EIF3D*, and *HNRNPA2B1* was able to independently predict the progression-free survival (Figures 6(g) and 6(h)). Therefore, *EIF3I*, *EIF3D*, and *HNRNPA2B1* can form a prognostic signature for prostate cancer relapse.

### 3.5. m^6^A-Dependent Functions of EIF3D and HNRNPA2B1 in Prostate Cancer

Given the prognostic importance of *EIF3D* and *HNRNPA2B1* for both overall and disease-free survival, we investigated their roles in prostate cancer.

**Figure 7:**
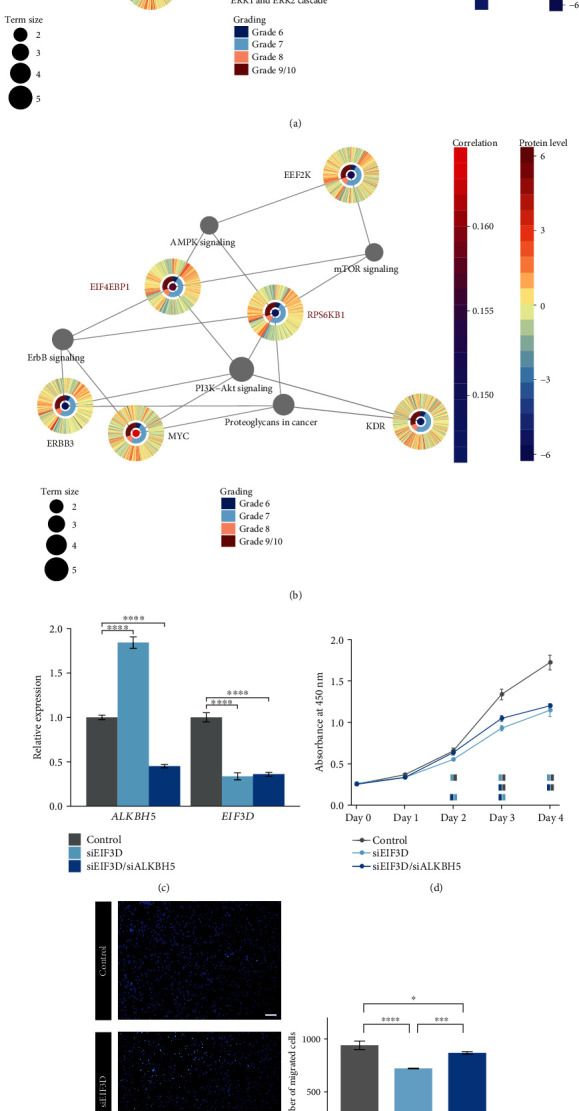
m^6^A-dependent function of EIF3D in prostate cancer. (a) Representative biological processes and (b) pathways in which the EIF3D induced proteins that participated in. Protein information is displayed in a form of a multiring circle, providing expression correlation (Pearson) with *EIF3D*, tumor grades, and *Z*-score-normalized protein levels from the inner to the outer layer. The outermost two layers are plotted in the way that each “spoke” represents a single sample, and samples are arranged in the same order for all proteins. The term size indicates the number of the EIF3D-induced proteins involved in a biological term. Edges connect genes and their involving terms. In addition, proteins subject to RNA m^6^A modifications are marked in red, including EIF4EBP1 and RPS6KB1. (c) mRNA level changes of *ALKBH5* and *EIF3D* in the human prostate cancer cell line C4-2B transfected with either *EIF3D* siRNAs only (siEIF3D) or *EIF3D* siRNAs plus *ALKBH5* siRNAs (siEIF3D/siALKBH5). (d) Effects of EIF3D and EIF3D/ALKBH5 knockdown on the growth curves of C4-2B cells. In each time point, control (grey blocks), EIF3D-knockdown (light blue blocks), and EIF3D/ALKBH5-knockdown (dark blue blocks) cells significantly differing in the viable cell number from each other are shown underneath the growth curves (*p* value < 0.05). Rows from the first to the last display comparisons of EIF3D-knockdown cells versus control, EIF3D/ALKBH5-knockdown cells versus control, and EIF3D/ALKBH5-knockdown cells versus EIF3D-knockdown ones, respectively. (e) Knockdown of EIF3D and EIF3D/ALKBH5 altered the migration ability of C4-2B cells. Left panel: representative micrographs of Transwell assays; right panel: pairwise comparisons of the migrated cell numbers between control, EIF3D-knockdown, and EIF3D/ALKBH5-knockdown cells. ∗ denotes *p* value < 0.05, ∗∗∗ denotes *p* value < 0.001, and ∗∗∗∗ denotes *p* value < 0.0001.

EIF3D is the cap-binding subunit of eIF3 [28] which mediates the translation initiation of m^6^A-containing mRNAs [14, 15]. Protein levels of EEF2K, EIF4EBP1, ERBB3, KDR, MYC, and RPS6KB1 exhibited a positive correlation with *EIF3D* in prostate cancer (Figures 7(a) and 7(b) and Supplementary Excel S6). Those six proteins might be under translational control of eIF3. Direct EIF3D binding was reported on the *ERBB3* transcripts in human HepG2 liver cancer cells [33] (Supplementary Excel S6). None of the six proteins was relevant to either death or relapse of prostate cancer except KDR which was a prognostic factor for improved recurrence-free survival according to the Kaplan–Meier survival analysis (*p* value = 0.051). Previous findings have revealed that EIF4EBP1 exerts an antioncogenic effect [34], ERBB3, MYC, and RPS6KB1 exert prooncogenic effects [35–37], and EEF2K plays a dual role [38]. The six proteins were implicated in translation (such as regulation of translation initiation and mTOR signaling), cell cycle, response to drug, and multiple signaling pathways (such as ERK/MEK cascade and PI3K-Akt signaling) (Figures 7(a) and 7(b) and Supplementary Excel S7). Moreover, EIF4EBP1 and RPS6KB1 showed a positive correlation with the catalytic subunit METTL3 in prostate cancer and contained candidate sites for m^6^A modifications (Supplementary Excel S6), suggesting m^6^A involvement in their translation.

**Figure 8:**
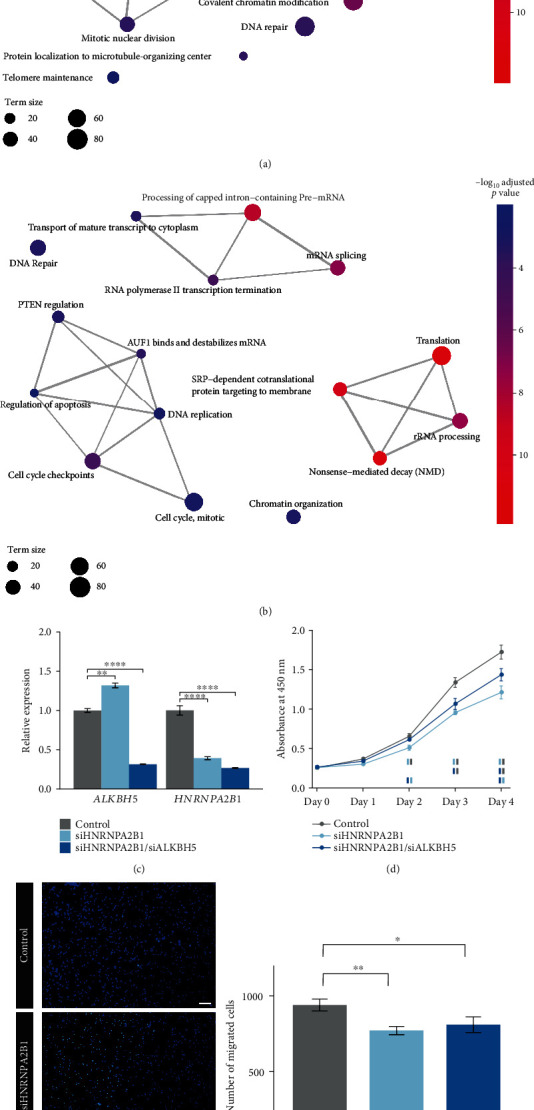
m^6^A-dependent function of HNRNPA2B1 in prostate cancer. (a) Representative biological processes and (b) pathways in which the m^6^A-dependent HNRNPA2B1 induced alternative splicing isoforms that participated in. The term size indicates the number of the m^6^A- and HNRNPA2B1-affected genes involved in a biological term. Edges connect overlapping terms and are scaled in thickness according to the number of shared genes. (c) Effects of siRNA transfection targeting either *HNRNPA2B1* (siHNRNPA2B1) or both *HNRNPA2B1* and *ALKBH5* (siHNRNPA2B1/siALKBH5) on mRNA levels of *ALKBH5* and *HNRNPA2B1* in human C4-2B prostate cancer cells. (d) Effects of HNRNPA2B1 and HNRNPA2B1/ALKBH5 knockdown on the growth curves of C4-2B cells. In each time point, control (grey blocks), HNRNPA2B1-knockdown (light blue blocks), and HNRNPA2B1/ALKBH5-knockdown (dark blue blocks) cells significantly differing in the viable cell number from each other are shown underneath the growth curves (*p* value < 0.05). Rows from the first to the last display comparisons of HNRNPA2B1-knockdown cells versus control, HNRNPA2B1/ALKBH5-knockdown cells versus control, and HNRNPA2B1/ALKBH5-knockdown cells versus HNRNPA2B1-knockdown ones, respectively. (e) Knockdown of HNRNPA2B1 and HNRNPA2B1/ALKBH5 altered the migration ability of C4-2B cells. Left panel: representative micrographs of Transwell assays; right panel: pairwise comparisons of the migrated cell numbers between control, HNRNPA2B1-knockdown, and HNRNPA2B1/ALKBH5-knockdown cells. ∗ denotes *p* value < 0.05, ∗∗ denotes *p* value < 0.01, and ∗∗∗∗ denotes *p* value < 0.0001.

Alarcon et al. first reported HNRNPA2B1 recognition of RNA m^6^A modifications based on CLIP-seq and RNase footprinting [39]. HNRNPA2B1 is involved in alternative splicing and primary miRNA processing, both of which rely on m^6^A patterns [39]. Here, we focused on the splicing function of HNRNPA2B1 in prostate cancer. A total of 1346 genes showed dependence of alternative splicing on both HNRNPA2B1 and m^6^A in prostate cancer (Supplementary Excel S8). Direct HNRNPA2B1 binding was reported on about 31% of those genes in breast cancer cells and breast epithelial cells [39–41] (Supplementary Excel S8). The affected genes participated in RNA processing (such as splicing, stability regulation, transport, and localization), cell cycle (such as spindle organization, chromosome segregation, nuclear division, and cell cycle checkpoints), DNA replication and repair, chromatin organization, translation, and mitochondrion-related processes (such as mitochondrial gene expression and transport) (Figures 8(a) and 8(b) and Supplementary Excel S9). Roles of HNRNPA2B1 in promoting cell proliferation have been reported in human embryonic stem cells [42] and diverse human cancer cell lines [43–46], such as regulating alternative splicing of tumor suppressors and oncogenes [43].

To determine the dependence of EIF3D and HNRNPA2B1 functions on the m^6^A modification, RNA interference of these two readers plus the eraser ALKBH5 was performed in the human prostate cancer cell line C4-2B. Transfection with siRNAs targeting either *EIF3D* or *HNRNPA2B1* effectively knocked down the target and not *ALKBH5*, and transfection with siRNAs targeting *ALKBH5* plus one of the two readers significantly decreased the expression of both targets (Figures 7(c) and 8(c)). Knockdown of EIF3D significantly decreased the cell proliferation rate (Figure 7(d)). Double knockdown of EIF3D and ALKBH5 also exhibited an inhibitory effect on cell proliferation, but 24 hours later than single knockdown of EIF3D (Figure 7(d)), which indicated that the m^6^A level might influence the function of EIF3D in cell proliferation. The same phenomena were observed in the knockdown of HNRNPA2B1 and HNRNPA2B1/ALKBH5 (Figure 8(d)), implying dependence of the HNRNPA2B1 role in promoting cell proliferation on the m^6^A pattern. Moreover, EIF3D knockdown significantly suppressed cell migration, but additional ALKBH5 knockdown reduced the inhibitory effect of EIF3D knockdown (Figure 7(e)). HNRNPA2B1 knockdown mildly decreased the cell migration ability, and additional ALKBH5 knockdown did not change the effect of HNRNPA2B1 knockdown (Figure 8(e)). Therefore, the m^6^A pattern might impact the function of EIF3D but not HNRNPA2B1 in cell migration.

## 4. Discussion

Roles of m^6^A regulatory components have been exemplified in cancer development [3, 4]. In this report, we systematically described molecular changes of the mRNA m^6^A machinery in primary prostate cancer and evaluated its clinical and survival significance. Somatic mutations of the m^6^A regulators were occasionally detected in prostate tumors. Although the degree of copy number aberrations was generally small, their effects were strong enough to alter the transcription levels of most m^6^A regulators. In fact, genomic changes of the m^6^A regulators are not frequent in other types of cancer either [18]. In addition, it is interesting to observe enrichment of CpG sites and extensive changes of DNA methylation in the m^6^A regulators across prostate tumors. Fine-tuning of transcription by CpG density alone [47, 48] and importance of the m^6^A machinery in development [1, 2] might explain the CpG enrichment. Impact of DNA methylation on the expression of the m^6^A regulators was weaker than that of the gene copy number. In primary prostate cancer, the majority of writers and readers were increased on the transcriptional level, while both erasers were decreased, indicating elevation of RNA m^6^A modifications which has been reported in a few prostate cancer cell lines and metastatic prostate tumors [30, 31]. Furthermore, it is not surprising to find that the m^6^A regulators of clinical and survival significance were largely readers because the readers determine the effects of m^6^A modifications [1–3]. But it did not always seem the same case in other cancer types [18]. For example, only ALKBH5 was relevant to the cholangiocarcinoma survival [18], which illustrates heterogeneity of the m^6^A system in tumors.

Relevance to clinical features and independent contribution to both overall and disease-free survival highlighted the importance of the translation initiation factor subunit EIF3D and the splicing factor HNRNPA2B1 in prostate cancer. Multiomics analysis revealed that EIF3D might prohibit the prostate cancer progression through translational control of translation, cell cycle, response to drug, and multiple signaling pathways. Due to the fact that only a few proteins have been assayed until now, it limits our analysis of the EIF3D function and its dependence on m^6^A in prostate carcinogenesis. However, *in vitro* studies showed that EIF3D knockdown suppressed prostate cancer cell proliferation and migration which were modulated by the m^6^A level. The contradiction might reflect the complexity of carcinogenesis, as exemplified by AR which generally drives proliferation of prostate cancer cells [49] but also induces DNA double-strand breaks and cell cycle arrest in the presence of supraphysiological androgens [50]. Another *in vitro* test demonstrated that HNRNPA2B1 promoted the proliferation of prostate cancer cells in an m^6^A-dependent manner, which might involve alternative splicing of genes participating in RNA processing, cell cycle, DNA replication and repair, chromatin organization, translation, and mitochondrion-related processes. HNRNPA2B1 has been shown to regulate alternative splicing of tumor suppressors and oncogenes in glioblastoma cell lines [43]. It should be noticed that there is still controversy around whether HNRNPA2B1 directly reads RNA m^6^A marks. A recent finding refuted the direct binding of HNRNPA2B1 to m^6^A modifications and proposed that m^6^A induced RNA unfolding which made RNAs more accessible [51].

## 5. Conclusions

In conclusion, thorough and systematic investigation depicted genomic, epigenomic, and transcriptomic features of the mRNA m^6^A machinery in primary prostate cancer. Furthermore, we revealed the potential cancer-relevant roles of two independent prognostic factors EIF3D and HNRNPA2B1 which may represent promising biomarkers for prostate cancer. The information provided by the present research may facilitate and spark further studies on the m^6^A machinery in prostate cancer initiation and progression.

## Data Availability

The datasets used in the present study are available from the corresponding authors upon reasonable request.

## Supplementary Materials

Supplementary Materials Supplementary materials include a word file, six figures, and nine spreadsheets. The supplementary materials.docx includes four tables and provides a description for each supplementary material file.
