# Supplementary material for: Systematic Investigation of mRNA N6-Methyladenosine Machinery in Primary Prostate Cancer
Source: Dis Markers. 2020 Nov 12;2020:8833438. doi: 10.1155/2020/8833438 (PMC7676945; doi:10.1155/2020/8833438)
Supplement: Supplementary Materials — include a word file, six figures, and nine spreadsheets. The supplementary materials.docx includes four tables and provides a description for each supplementary material file. [file 8833438.f1.zip › 8833438.f1/supplementary materials.docx]

**Supplementary Figures and Tables**

**Figure S1** More CpG methylation sites were observed in the mRNA m^6^A regulators in prostate cancer, including promoters **(A)**, upstream distal regulatory regions **(B)**, and gene bodies **(C)**

**Figure S2**  Genomic methylation changes of the mRNA m^6^A regulators in prostate cancer. **(A–C)** Differential levels of CpG methylation between prostate cancer and normal prostate in writers, erasers, and readers respectively. * denotes *p*-value < .05; ** denotes *p*-value < .01; *** denotes *p*-value < .001; **** denotes *p*-value < .0001

**Figure S3**  Differential expression of the mRNA m^6^A regulator genes in prostate cancer. Writers, erasers, and readers are marked in orange, green, and grey respectively. * denotes *p*-value < .05; ** denotes *p*-value < .01; *** denotes *p*-value < .001; **** denotes *p*-value < .0001

**Figure S4**  Differential expression of the mRNA m^6^A regulator genes among histologic grades of prostate cancer. * denotes *p*-value < .05; ** denotes *p*-value < .01; *** denotes *p*-value < .001; **** denotes *p*-value < .0001. G6, G7, G8, and G9/10 are short for Grade 6, Grade 7, Grade 8, and Grade 9/10 respectively

**Figure S5** Varied expression of the mRNA m^6^A regulator genes among pathologic stages of prostate cancer. **(A)** Differentially expressed regulator genes among T stages. **(B)** Differentially expressed regulator genes between N stages. * denotes *p*-value < .05; ** denotes *p*-value < .01; *** denotes *p*-value < .001; **** denotes *p*-value < .0001

**Figure S6**  Differential expression of the mRNA m^6^A regulator genes among molecular subtypes of prostate cancer. * denotes *p*-value < .05; ** denotes *p*-value < .01; *** denotes *p*-value < .001; **** denotes *p*-value < .0001

**Table S1** Sequences of Synthetic siRNA Oligonucleotides

| Target | Sense | Antisense |
| --- | --- | --- |
| Negative Control | UUCUCCGAACGUGUCACGUTT | ACGUGACACGUUCGGAGAATT |
| *ALKBH5* | UCAACAGCGCCGUCAUCAATT | UUGAUGACGGCGCUGUUGATT |
| *EIF3D* | CCAGAGAGUUGGGUCCAAATT | UUUGGACCCAACUCUCUGGTT |
| *HNRNPA2B1* | GGAACAUCACCUUAGAGAUTT | AUCUCUAAGGUGAUGUUCCTT |

**Table S2** Sequences of Primers Used in the Quantitative RT-PCR

| Target | Forward Primer | Reverse Primer |
| --- | --- | --- |
| *Actin* | CCTCTCCCAAGTCCACACAG | GGGCACGAAGGCTCATCATT |
| *ALKBH5* | CGTGACTGTGCTCAGTGGAT | GAGCTGCTCAGGGACTTTGT |
| *EIF3D* | TTTGCCACTGATGCCATCCT | CACTGTCAGGAGGTCAAAGTCA |
| *HNRNPA2B1* | GAGTCCGCGATGGAGAGAGA | TGCTTGCAGGATCCCTCATT |

**Table S3** Members of the mRNA m^6^A Machinery

| Symbol | Entrez ID | Ensembl ID | Class | References^a^ |
| --- | --- | --- | --- | --- |
| METTL3 | 56339 | ENSG00000165819 | Writer | [1-3] |
| METTL14 | 57721 | ENSG00000145388 | Writer | [1-3] |
| CBLL1 | 79872 | ENSG00000105879 | Writer | [2] |
| RBM15 | 64783 | ENSG00000162775 | Writer | [1-3] |
| RBM15B | 29890 | ENSG00000259956 | Writer | [1-3] |
| VIRMA | 25962 | ENSG00000164944 | Writer | [1-3] |
| WTAP | 9589 | ENSG00000146457 | Writer | [1-3] |
| ZC3H13 | 23091 | ENSG00000123200 | Writer | [2] |
| METTL16 | 79066 | ENSG00000127804 | Writer | [1-3] |
| TRA2A | 29896 | ENSG00000164548 | Writer | [4] |
| CAPRIN1 | 4076 | ENSG00000135387 | Writer | [4] |
| ALKBH5 | 54890 | ENSG00000091542 | Eraser | [1-3] |
| FTO | 79068 | ENSG00000140718 | Eraser | [1-3] |
| EIF3A | 8661 | ENSG00000107581 | Reader | [1-3] |
| EIF3B | 8662 | ENSG00000106263 | Reader | [1-3] |
| EIF3C | 8663 | ENSG00000184110 | Reader | [1-3] |
| EIF3D | 8664 | ENSG00000100353 | Reader | [1-3] |
| EIF3E | 3646 | ENSG00000104408 | Reader | [1-3] |
| EIF3F | 8665 | ENSG00000175390 | Reader | [1-3] |
| EIF3G | 8666 | ENSG00000130811 | Reader | [1-3] |
| EIF3H | 8667 | ENSG00000147677 | Reader | [1-3] |
| EIF3I | 8668 | ENSG00000084623 | Reader | [1-3] |
| EIF3J | 8669 | ENSG00000104131 | Reader | [1-3] |
| EIF3K | 27335 | ENSG00000178982 | Reader | [1-3] |
| EIF3L | 51386 | ENSG00000100129 | Reader | [1-3] |
| EIF3M | 10480 | ENSG00000149100 | Reader | [1-3] |
| FMR1 | 2332 | ENSG00000102081 | Reader | [2, 3] |
| HNRNPA2B1 | 3181 | ENSG00000122566 | Reader | [1-3] |
| HNRNPC | 3183 | ENSG00000092199 | Reader | [1-3] |
| IGF2BP1 | 10642 | ENSG00000159217 | Reader | [2, 3] |
| IGF2BP2 | 10644 | ENSG00000073792 | Reader | [2] |
| IGF2BP3 | 10643 | ENSG00000136231 | Reader | [2] |
| LRPPRC | 10128 | ENSG00000138095 | Reader | [3] |
| PRRC2A | 7916 | ENSG00000204469 | Reader | [5] |
| PRRC2C | 23215 | ENSG00000117523 | Reader | [5] |
| RBMX | 27316 | ENSG00000147274 | Reader | [6] |
| YTHDC1 | 91746 | ENSG00000083896 | Reader | [1-3] |
| YTHDC2 | 64848 | ENSG00000047188 | Reader | [1-3] |
| YTHDF1 | 54915 | ENSG00000149658 | Reader | [1-3] |
| YTHDF2 | 51441 | ENSG00000198492 | Reader | [1-3] |
| YTHDF3 | 253943 | ENSG00000185728 | Reader | [1-3] |

a References which reported the gene functions in mRNA m^6^A modifications

**Table S4** Mutations in mRNA m^6^A Readers, Writers, and Erasers

| Gene | Sample Number^a^ | Mutation Type |
| --- | --- | --- |
| *METTL3* | 1 | Missense |
| *METTL14* | 1 | Missense |
| *CBLL1* | 1 | Missense |
| *RBM15* | 1 | Frame Shift Del |
| *RBM15B* | 1 | Missense |
| *WTAP* | 1 | Missense |
| *ZC3H13* | 6 | Nonsense, Missense, Frame Shift Del |
| *TRA2A* | 1 | Splice Site |
| *CAPRIN1* | 1 | Missense |
| *ALKBH5* | 1 | Missense |
| *EIF3B* | 2 | Missense, Missense |
| *EIF3C* | 1 | Missense |
| *EIF3D* | 1 | Missense |
| *EIF3F* | 1 | Missense |
| *EIF3J* | 1 | Missense |
| *EIF3L* | 1 | Nonsense |
| *FMR1* | 1 | Missense |
| *HNRNPC* | 1 | Missense |
| *IGF2BP2* | 1 | Missense |
| *PRRC2A* | 3 | Nonsense, Missense, In Frame Del |
| *PRRC2C* | 1 | Missense |
| *RBMXL1* | 1 | Missense |
| *YTHDC1* | 1 | Frame Shift Del |
| *YTHDC2* | 2 | Missense, Nonsense |
| *YTHDF1* | 1 | Missense |
| *YTHDF2* | 1 | Missense |

a Number of samples carrying the indicated mutations

**References**

1 Meyer KD, Jaffrey SR. Rethinking m(6)A Readers, Writers, and Erasers. *Annu Rev Cell Dev Biol* 2017; 33: 319-342.

2 Zaccara S, Ries RJ, Jaffrey SR. Reading, writing and erasing mRNA methylation. *Nat Rev Mol Cell Biol* 2019; 20: 608-624.

3 Chen XY, Zhang J, Zhu JS. The role of m(6)A RNA methylation in human cancer. *Mol Cancer* 2019; 18: 103.

4 An S, Huang W, Huang X, Cun Y, Cheng W, Sun X *et al*. Integrative network analysis identifies cell-specific trans regulators of m6A. *Nucleic Acids Res* 2020; 48: 1715-1729.

5 Wu R, Li A, Sun B, Sun JG, Zhang J, Zhang T *et al*. A novel m(6)A reader Prrc2a controls oligodendroglial specification and myelination. *Cell Res* 2019; 29: 23-41.

6 Zhou KI, Liu N, Pan T. Identification of N(6)-methyladenosine reader proteins. *Methods* 2017; 126: 105-111.

**Excel S1**  Genomic methylation changes of the mRNA m^6^A regulators in prostate cancer

**Excel S2**  Differential expression of the mRNA m^6^A regulator genes in prostate cancer

**Excel S3**  Expression variations of the mRNA m^6^A regulator genes among prostate tumor grades

**Excel S4**  Expression variations of the mRNA m^6^A regulator genes among prostate tumor stages

**Excel S5**  Expression variations of the mRNA m^6^A regulator genes among molecular subtypes of prostate cancer

**Excel S6**  EIF3D induced proteins

**Excel S7**  Biological processes and pathways enriched in the EIF3D induced proteins

**Excel S8**  m^6^A-dependent HNRNPA2B1 induced alternative splicing isoforms

**Excel S9**  Biological processes and pathways enriched in the m^6^A-dependent HNRNPA2B1 induced alternative splicing isoforms
