# Supplementary figures and images for: Systematic Investigation of mRNA N6-Methyladenosine Machinery in Primary Prostate Cancer
Source: Dis Markers. 2020 Nov 12;2020:8833438. doi: 10.1155/2020/8833438 (PMC7676945; doi:10.1155/2020/8833438)

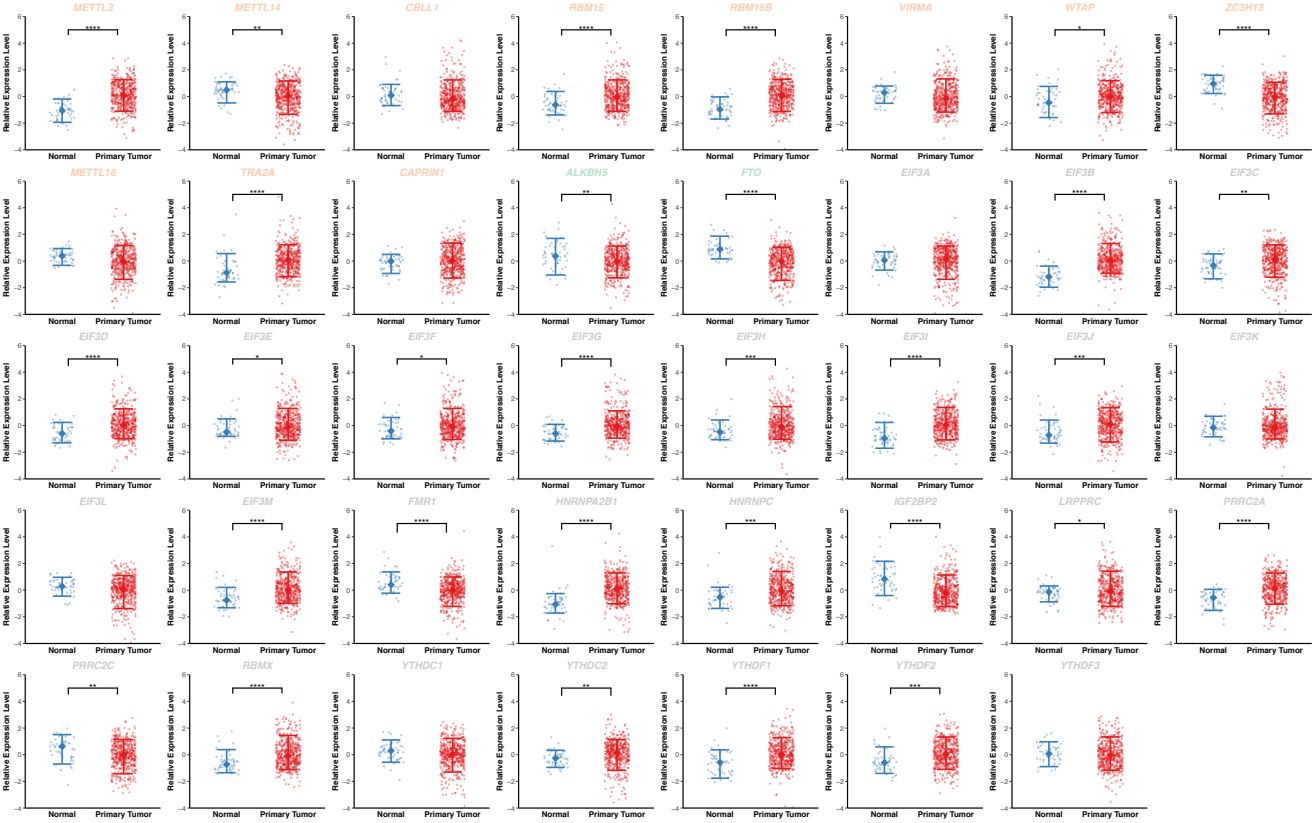

Supplement: Supplementary Materials — include a word file, six figures, and nine spreadsheets. The supplementary materials.docx includes four tables and provides a description for each supplementary material file. [file 8833438.f1.zip › 8833438.f1/Fig S3 (1).pdf]

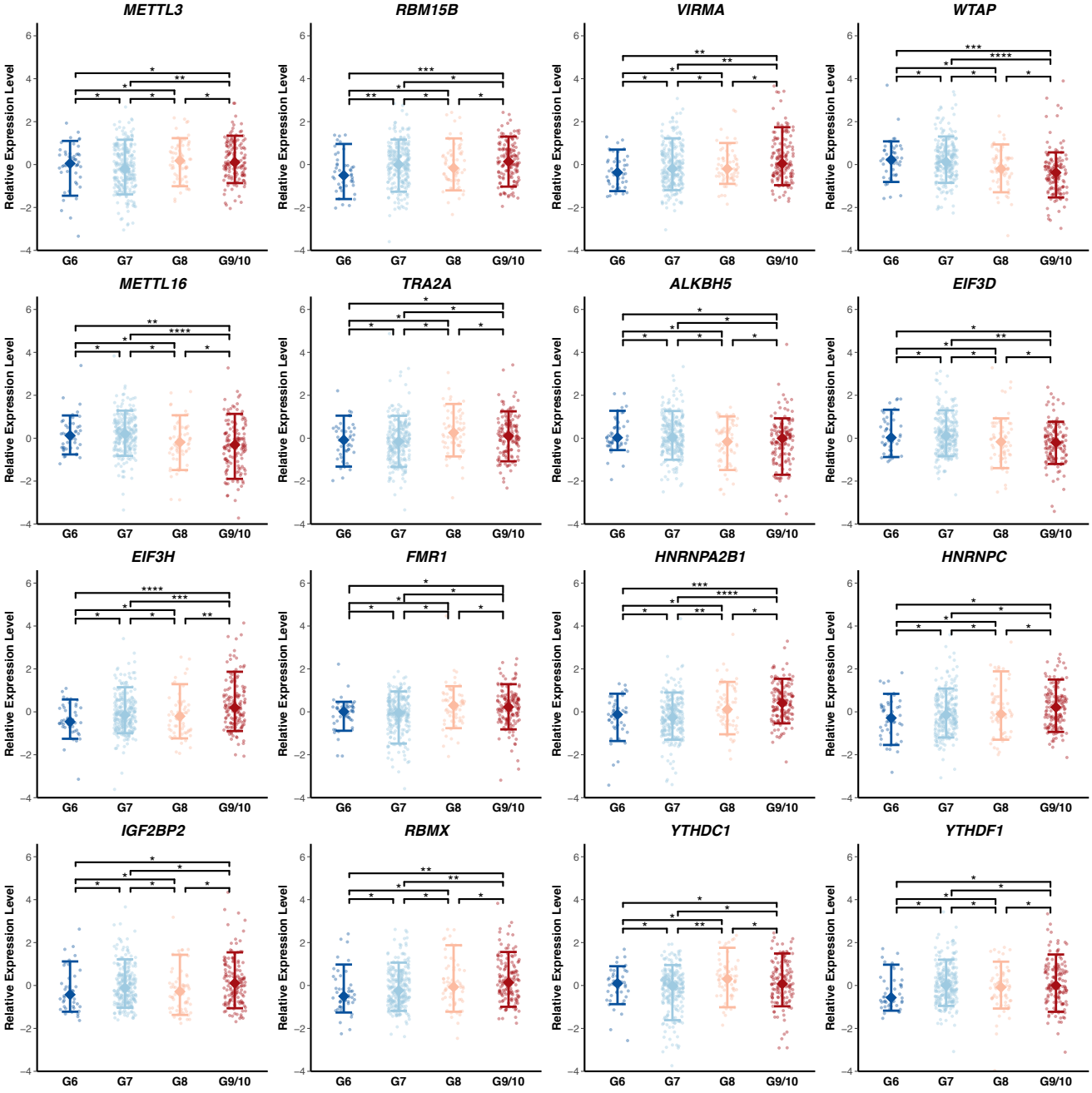

Supplement: Supplementary Materials — include a word file, six figures, and nine spreadsheets. The supplementary materials.docx includes four tables and provides a description for each supplementary material file. [file 8833438.f1.zip › 8833438.f1/Fig S4 (1).pdf]

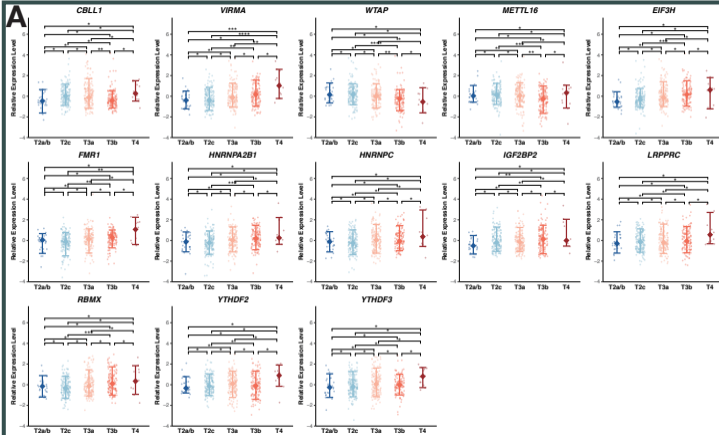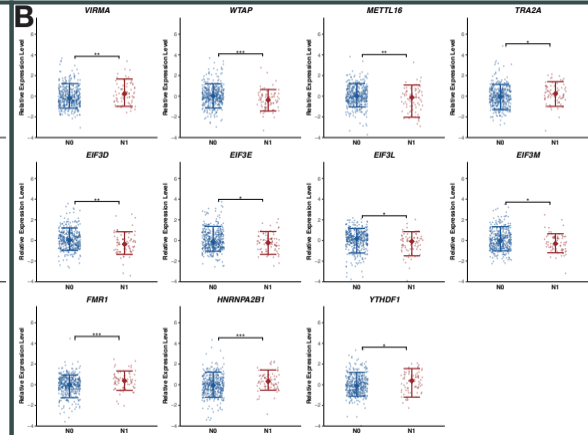

Supplement: Supplementary Materials — include a word file, six figures, and nine spreadsheets. The supplementary materials.docx includes four tables and provides a description for each supplementary material file. [file 8833438.f1.zip › 8833438.f1/Fig S5 (1).pdf]
