# Supplementary material for: Systematic Investigation of mRNA N6-Methyladenosine Machinery in Primary Prostate Cancer
Source: Dis Markers. 2020 Nov 12;2020:8833438. doi: 10.1155/2020/8833438 (PMC7676945; doi:10.1155/2020/8833438)
Supplement: Supplementary Materials — include a word file, six figures, and nine spreadsheets. The supplementary materials.docx includes four tables and provides a description for each supplementary material file. [file Fig S6 (1).pdf › 8833438.f1/Fig S6 (1).pdf]

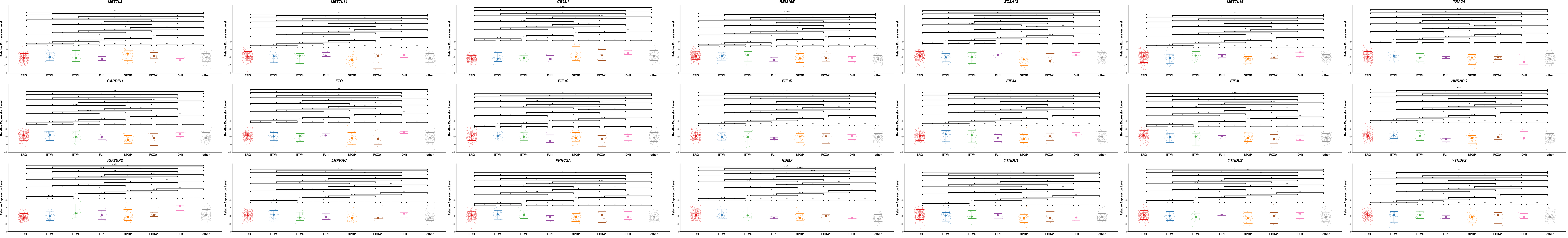
METTL3
METTL14
CBLL1
RBM15B
ZC3H13
METTL16
TRA2A
CAPRIN1
FTO
EIF3C
EIF3D
EIF3J
EIF3L
HNRNPC
IGF2BP2
LRPPRC
PRRC2A
RBMX
YTHDC1
YTHDC2
YTHDF2
Relative Expression Level
ERG
ETV1
ETV4
FLI1
SPOP
FOXA1
IDH1
other
